# RNA Binding Proteins and Genome Integrity

**DOI:** 10.3390/ijms18071341

**Published:** 2017-06-23

**Authors:** Kensei Nishida, Yuki Kuwano, Tatsuya Nishikawa, Kiyoshi Masuda, Kazuhito Rokutan

**Affiliations:** 1 Department of Pathophysiology, Institute of Biomedical Sciences, Tokushima University Graduate School, Tokushima 770-8503, Japan; kuwanoy@tokushima-u.ac.jp (Y.K.); nishikawa.tatsuya@tokushima-u.ac.jp (T.N.); rokutan@tokushima-u.ac.jp (K.R.); 2 Department of Human Genetics, Institute of Biomedical Sciences, Tokushima University Graduate School, Tokushima 770-8503, Japan; kiyoshim@tokushima-u.ac.jp

**Keywords:** RNA-binding protein, R-loop, telomere, DNA damage response

## Abstract

Genome integrity can be threatened by various endogenous or exogenous events. To counteract these stressors, the DNA damage response network contributes to the prevention and/or repair of genomic DNA damage and serves an essential function in cellular survival. DNA binding proteins are involved in this network. Recently, several RNA-binding proteins (RBPs) that are recruited to DNA damage sites have been shown to be direct players in the prevention or repair of DNA damage. In addition, non-coding RNAs, themselves, are involved in the RNA-mediated DNA repair system. Furthermore, RNA modification such as m6A methylation might also contribute to the ultraviolet-responsive DNA damage response. Accumulating evidence suggests that RNA metabolism is more deeply involved in diverse cellular functions than previously expected, and is also intricately associated with the maintenance of genome integrity. In this review, we highlight the roles of RBPs in the maintenance of genome integrity.

## 1. Introduction

Genome integrity is a fundamental issue that is associated with cell survival. To counteract stress-induced DNA damage such as aberrant DNA replication, transcription, and uncontrolled cell division, the coordination of numerous proteins is required to maintain genome integrity for organism survival. Recently, RNA-binding proteins (RBPs) have emerged as important players and coordinators in the maintenance of genome integrity. RBPs preferentially bind mature mRNA sequences; therefore, RBPs are considered to regulate diverse aspects of mRNA metabolism and modulate the destiny of mRNAs. [1]. Once RNA molecules are transcribed, RBPs associate with nascent transcripts to form messenger ribonucleoprotein (mRNP) complexes. Co-transcriptional assembly of these mRNPs modulates post-transcriptional processes, including polyadenylation, splicing, localization, stabilization, and translation effifciency of mRNAs. RBPs can also contribute to essential functions in non-coding RNA (ncRNA) biogenesis (e.g., microRNAs (miRNA)) [2]. RBPs and miRNAs cooperatively modulate of the post-transcriptional regulatory network during the maintenance of cellular homeostasis. Given the central role of RBPs in the regulation of mRNA fate, dysfunctional RBPs can lead to various disease pathologies including neurodegenerative disorders, cardiovascular diseases, and cancers [1,3,4,5]. Gerstberger et al*.* presented an extensive classification of the 1542 RBPs in humans, representing 7.5% of all protein-coding genes [1]. Whereas approximately half of these RBPs can be grouped based on their mRNA targets, others might interact with a variety RNAs including transfer RNAs, small nuclear RNAs, small nucleolar RNAs, or ncRNAs [1]. In addition to binding RNA species, RBPs can interact with various functional proteins, suggesting undiscovered roles in cellular processes including the maintenance of genome integrity. Interestingly, it is known that some of RBPs can bind DNA as well as RNA species. In other words, some DNA-binding proteins have the capability to bind RNA. Hudson et al. defined 407 DNA- and RNA-binding proteins (DRBPs) among 1267 RNA-binding proteins. Ontology analysis of DRBPs indicated that many of these might be involved in unexpected biological processes including the DNA-damage response (DDR), apoptosis, and responses to extreme temperature [6]. Accumulating knowledge suggests that the functional domains of proteins with both DNA- and RNA-binding capacity are important for the modulation of cellular homeostasis, including gene expression, cell proliferation, DDR, and genome stability. In addition, crucial roles for RBPs in the modulation of R-loop formation have been noted to be highly important for genome integrity [7]. Moreover, advanced analytical techniques such as RNA deep sequencing and single cell analysis have been providing us with unexpected functions of RBPs and RNA species in genome maintenance. Given the accelerating pace of this field, comprehensive reviews regarding RBPs- and RNA-mediated genome maintenance will be useful. Thus, in this review, we focus on aspects of the genome maintenance function of RBPs, especially regarding R-loop formation, telomerase activity, DNA damage response, and replication.

## 2. Role of RBPs in R-Loop Formation

**Figure 1:**
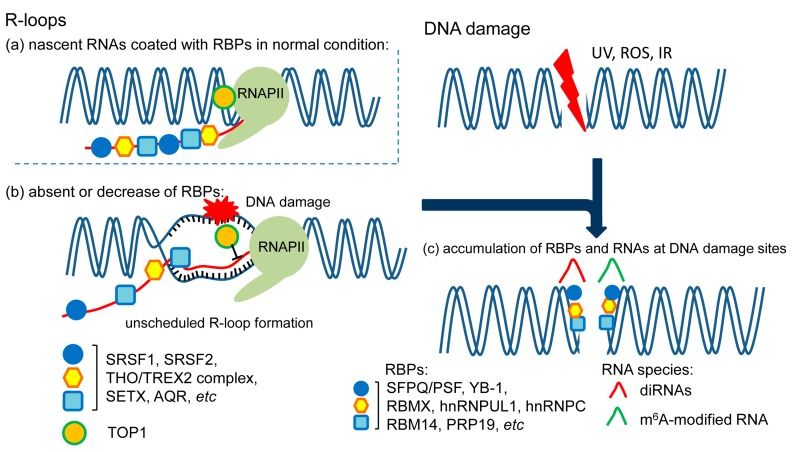
Roles of RNA-binding proteins (RBPs) in R-loops formation and DNA damage response. (**a**) RBPs coat nascent RNAs and prevent transcription-associated DNA:RNA hybrids (R-loops). Topoisomerase (TOP1) resolves the local negative supercoils behind the transcribing RNA polymerase II (RNAPII); (**b**) R-loop formation is accumulated in cells with absent or decrease of RBPs, inducing DNA damage at the non-template single-strand DNA; (**c**) various stressors, such as ultraviolet (UV), reactive oxygen species (ROS), and ionizing radiation (IR) evoke DNA damage. Certain RBPs and RNA species are recruited at DNA damage sites. RNA species serve as sensors of DNA damage or templates in DNA repair process, and RBPs interact with DNA repair proteins and facilitate DNA damage responses.

DNA:RNA hybrids form during normal transcription and replication. The hybrids (8-bp DNA:RNA duplexes), which form inside the active site of the RNA polymerase, are considered as the normal transcription bubble. During replication, ~11-nt-long RNA primers form the hybrids with the DNA templates [8]. In contrast to these relatively short hybrids, a longer form of DNA:RNA hybrid is known as an R-loop. The nascent RNA hybridizes with the template single-stranded DNA (ssDNA), leading to the formation of a DNA:RNA hybrid. The DNA:RNA hybrid on the template strand and the associated non-template ssDNA form three-stranded nucleic acid structures, which is the R-loop [7] (Figure 1a,b). Since the first identification of R-loops in bacteria [9], they have been found in many organisms, and have been observed in greater than 5% of the human genome [10]. R-loops usually have short life spans (approximately 20 min), and are efficiently removed under normal cellular conditions. The programmed formation of R-loops has an important role in diverse cellular processes, such as transcriptional termination [11], mitochondrial DNA replication [12], immunoglobulin class switching [13], and chromatin modifications [10,14]. However, unscheduled (stable) R-loops might represent a major threat to genome integrity. There are two major sources of R-loop formation: one is polymerase collisions between replication forks and transcription elongation machinery, whereas the other is a lack of RBPs coating the nascent RNAs. For example, if R-loops are not properly eliminated, conflicts between unscheduled R-loops and replication forks can increase the risk of compromised genome integrity [15]. When there is a replication-transcription conflict, the replication fork always loses the replisome due to the stability of the R-loop resulting from the conflict-induced RNA polymerase stalling. The stability of replication forks may be mediated by the resulting R-loop formation. As a consequence of the conflict, whereas the DNA:RNA hybrid structures of R-loops are particularly stable due to thermodynamic stability, the non-template ssDNA is relatively unstable and susceptible to transcription-associated mutagenesis and recombination [16,17,18]. Certain regions of the genome, where R-loops that are associated with ssDNA accumulate, can become more chemically labile, leading to the accumulation of proteins that recognize R-loop structures, such as DNA-modifying enzymes or other repair factors. Some of these proteins might be involved in initiating mutagenesis. Alternatively, collisions between transcription and replication complexes induce replication-fork stalling, leading to impaired DNA replication which, in turn, results in DNA double-stranded breaks through fork collapse or the initiation of mitosis with incomplete DNA replication [19,20] (Figure 1).

However, in normal cells, distinct protection mechanisms usually overcome the formation of these R-loops and mitigate their detrimental effects to preserve genome integrity. These regulatory proteins, found during R-loop formation, can be categorized into two major subgroups based on their function: (1) factors that prevent R-loop formation such as DNA topoisomerase I, SRSF1, and the THO/TREX complex; and (2) factors that remove R-loops, such as RNase H enzymes and RNA/DNA helicases [15] (representative lists of regulatory genes are reviewed in [7,20]).

DNA topoisomerase I, an evolutionarily-conserved regulator of DNA repair and transcription, suppresses R-loop-mediated genome instability through relaxing RNA polymerase-generated negative DNA supercoils. Defects or dysfunctions in these regulators accumulate aberrant R-loops formation in various cell types including *Saccharomyces cerevisiae*, mouse, and human cells [21,22].

RBPs are considered to prevent the formation of RNA:DNA hybrid R-loop structures by coating the nascent RNA. For example, R-loops were shown to accumulate in serine-arginine (SR) rich protein 1 (SRSF1)-depleted cells. SRSF1 is a well-studied multifunctional SR protein that plays important roles in mRNA splicing, localization, stability, and translation [23]. Li and Manley performed a genetic screen of SRSF1-depleted cells and revealed an unexpected function of SRSF1, specifically the maintenance of genome integrity [24,25]. SRSF1 absence induced a hypermutagenic phenotype due to an accumulation of R-loop structures, which can be converted into double-strand breaks (DSBs) by the transcription-coupled nucleotide excision repair factor Cockayne syndrome group B [26]. SRSF2 and SRSF3 were also shown to prevent R-loop formation, suggesting that the recruitment of SR proteins, through the splicing of introns on the nascent RNA during transcription, prevents aberrant RNA:DNA hybrid structures and undesired DSBs.

The THO/TREX-2 complex plays a central role in the packaging of nascent RNAs with various RBPs. Indeed, dysfunction of THO/TREX2 affects mRNA processing including transcriptional elongation and mRNA export [27,28]. Mutants of *HRP1* and *THO2*, encoding components of THO, result in a transcription-associated hypermutation phenotype in yeast [29,30]. A subsequent study revealed that this phenotype is directly associated with aberrant R-loop formation [31]. In addition, the yeast Npl3, the most abundant heterogeneous nuclear ribonucleoprotein (hnRNP), also prevented R-loop stabilization [32], suggesting that mRNA metabolism especially that associated with transcription, is deeply involved in the maintenance of genome integrity.

The RNA/DNA helicase Senataxin (SETX) is involved in the regulation of R-loop formation [26]. In normal biology, R-loop formation is required for proper transcriptional termination (RNA pol II pausing), and especially for RNA pol II-driven genes with G-rich pause sites [33]. Sen1, the yeast homolog of human SETX, was shown to suppress unscheduled R-loop formation and help to release RNA molecules from the transcriptional termination complex, which includes R-loop structures [34,35]. Another RNA/DNA helicase named Aquarius (AQR) might also be involved in the removal of R-loops [26].

RNase H enzymes have been reported as direct regulators of this process, as they degrade the RNA moiety of DNA:RNA hybrids. Depletion of endogenous RNase H activity results in the failure to remove R-loops in *Saccharomyces cerevisiae*, causing DNA damage that is preferentially observed in the repetitive ribosomal DNA locus [36]. Furthermore, Ohle et al. demonstrated that a direct evidence of a role of DNA:RNA hybrids involved in DNA damage repair. RNase H enzymes play an essential role in eliminating DSB-induced DNA:RNA hybrids, leading to the efficient repair of DSBs in yeast [37]. It has been suggested that RNase H enzymes might be involved in modulating the fate of a variety DNA:RNA hybrids including R-loop formation and DNA repair process.

Recently, the maintenance of R-loop homeostasis is considered to be associated with genome integrity and to be a dynamic process that counteracts deleterious consequences during transcription or replication. Furthermore, aberrant R-loop formation is an important contributor to human diseases [38,39]. Comprehensive studies on the mechanisms that regulate R-loop homeostasis are needed to understand human diseases and to develop novel therapeutic strategies in the future.

## 3. Telomere Shortening

**Figure 2:**
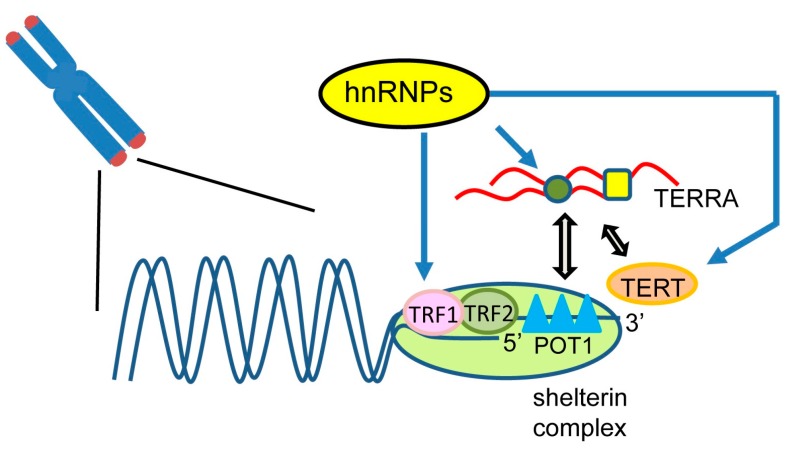
Roles of RNA-binding proteins (RBPs) in telomere activity. Heterogeneous nuclear proteins (hnRNPs) play multifunctional roles in regulating telomere maintenance. The shelterin complex, including telomeric repeat binding factor-1 (TRF1), TRF2, and protection of telomeres-1 (POT1), regulate the maintenance of telomere length. Telomeric repeat-containing RNA (TERRA) localizes to telomeres and regulates telomerase activity and telomere length.

Telomeres play important roles in protecting chromosomal ends from DNA damage during the maintenance of genome stability. Shortening of telomeres leads to detrimental cellular changes, such as inhibition of cell division and increased cellular senescence [40,41] (Figure 2). Recent studies have shown that telomere attrition influences mortality in inherited telomere aging-related diseases [42,43]. In addition, variations in telomere maintenance affect cancer progression [44]. Activation of telomerase overcomes telomere shortening, resulting in sustained cell replication during malignant transformation [45]. Telomere maintenance is mainly regulated by three factors: a tract of tandemly-repeated DNA sequences, associated protein, such as shelterin, and the telomerase complex [46]. A single-stranded G-rich overhang is formed through strand invasion of the 3’ overhang at the telomere end, which is called the telomere loop (t-loop). Because of exonuclease degradation after DNA replication and inabilities of DNA polymerases, human telomeres are shortened by ~50 base pairs per cell division. The shortened telomeres are reconstructed by telomerase reverse transcriptase (TERT), which can add TTAGGG repeats to the 3′ DNA ends of the chromosome [43,46]. Alterations in the expression of telomere-associated proteins are closely related to telomere dysfunction, which trigger chromosomal instability and tumorigenesis. Here, we focus on novel roles of RBPs in the modulation of regulatory factors required for telomere maintenance.

## 4. Roles of Heterogeneous Nuclear Ribonucleoproteins (hnRNP) in Telomere Regulation

Increasing evidence suggests that several RBPs, especially hnRNPs, are involved in the regulation of telomeres and the maintenance of genome integrity (Figure 2). As shown in Figure 2, hnRNPs are implicated in the control of telomere activity at multiple steps. We categorized telomere-related RBPs into four groups by their functions: (1) modulating associations among components of the telomeric complexes including telomeric DNA; (2) controlling localization of functional non-coding RNAs such as TERRA; (3) directly binding to TERT; and (4) transcriptionally or post-transcriptionally regulating gene expression of telomerase regulatory factors such as telomeric DNA-binding proteins.

The first group of RBPs contains hnRNP A1, fused-in-sarcoma (FUS), and telomeric repeat-binding factor (TRF) 1 and 2. hnRNP A1, a regulator of pre-mRNA splicing, binds telomeric DNA and enhances telomerase activity by altering G-rich overhang structures [47]. hnRNP A1 also interacts with a long non-coding telomeric repeat-containing RNA (TERRA), which is transcribed from telomere DNA. This complex regulates telomere coating by POT1, one of the shelterin complex proteins, during DNA replication [48]. FUS, also known as translocated in liposarcoma (TLS), interacts with the G-quadruplex, consisting of telomere DNA and TERRA, via its Arg-Gly-Gly repeat (RGG) domains to regulate histone modifications of telomeres [49,50]. TRF1 is a component of the shelterin protein complex that regulates telomere length by modulating the accessibility of telomerase to telomeres [51]. Therefore, the expression of TRF1 needs to be strictly controlled to ensure sufficient telomere function. A pre-mRNA splicing factor, U2 small nuclear ribonucleoprotein (snRNP) auxiliary factor 65 (U2AF65), interacts with TRF1 and stabilizes TRF1 by inhibiting its ubiquitin-dependent regulation [52]. TRF2 is also a member of the shelterin protein complex, which functions in telomere protection [45]. Inhibition of TRF2 triggers a DNA damage response and affects cell fate in neuronal cells [53].

The second group of RBPs contains hnRNP A/B, hnRNP F, and hnRNP A1. hnRNP A/B and hnRNP F bind to TERRA to control its abundance and localization, which influences telomere lengthening [54]. hnRNP A1 is already mentioned above.

The third group of RBPs contains hnRNP C and hnRNP U. Through the affinity purification of endogenous telomerase complexes, hnRNP C and hnRNP U were suggested to associate with TERT and influence telomere shortening [55].

The last group of RBPs contains AUF1, hnRNP H1 and H2, Yra1, and hnRNP A18. AUF1 (also referred to as hnRNP D), a RNA destabilizing factor of AU-rich element-containing mRNAs, is a regulator of telomere maintenance. AUF1 binds to the promoter of the mouse TERT (mTert) to activate its transcription. AUF1-knockout mice exhibited rapid premature aging caused by significant telomere erosion and pronounced cellular senescence [56]. hnRNP H1 and H2 specifically interact with exon 7 of Trf2 pre-mRNA and regulate alternative splicing of Trf2 mRNA, which is implicated in neuronal differentiation [57]. Yra1 is a member of the hnRNP-like family, which is involved in the regulation of mRNA export and cell growth [58]. Gavaldá et al*.* showed that Yra1 overexpression in yeast results in impaired DNA replication and a cellular senescence-like phenotype, by altering associations between Rrm3 DNA helicase and telomeres [59]. A recent study reported that hnRNP A18, also called cold inducible RNA-binding protein, interacts with telomerase and maintains telomerase activities in a temperature-dependent manner. Inhibition of hnRNP A18 reduces telomerase activities and shortens telomeres [60]. Thus, these data demonstrate the possible roles of RBPs in diverse cellular processes that are involved telomere regulation, which tightly regulate the preservation of genome integrity.

## 5. Roles of RBPs in DNA Damage Responses

Various environmental and chemical agents or cell-derived stressors, such as ionizing radiation, ultraviolet light, or reactive oxygen species, continually evoke DNA damage, such as DSBs. To ensure genome integrity, damaged cells upregulate a signaling network known as the DNA damage response (DDR). In general, DDR signaling is orchestrated by three PI3K-like protein kinases, specifically ATM (ataxia-telangiectasia mutated), ATR (ATM and Rad3-related), and DNA-PK (DNA-dependent protein kinase). The activation of these kinases facilitates the accumulation of proteins involved in DNA repair and chromatin modification or remodeling [61]. Recently, new functions for RBPs in the DDR have been described, and increasing evidence has suggested a role in the DNA repair process. Whereas some RBPs permit the selective expression of DDR genes in response to DNA damage through post-transcriptional regulation, some specific RBPs directly bind to sites of DNA damage and interact with DNA and repair proteins. Many reports and reviews have already demonstrated that DNA damage facilitate RBP-mediated post-transcriptional regulation of DDR genes [62,63,64]. Thus, here we discuss the specific RBPs that accumulate at sites of DNA damage (Figure 1c).

It is thought that DNA damage stimulates a distinct set of enzymes that facilitates DNA repair signaling. Recently, several studies have shown that some RBPs localized to sites of DNA damage, and they can directly interact with DNA or repair proteins [65,66,67,68,69,70,71,72,73]. For example, the DNA/RNA-binding nucleocytoplasmic shuttling protein, YB-1, was first described as a cytoplasmic mRNP that regulates mRNA translation, stability, and storage. In addition to mRNA metabolism, YB-1 appears to play a role in DNA repair by interacting with the DNA duplex and DNA repair proteins. YB-1 also functions in strand separation and has endonuclease activity in vitro [74,75,76].

Paraspeckles are sub-nuclear bodies composed of RNA-protein structures including ncRNAs and core proteins, such as non-POU domain-containing octamer-binding protein (NONO), splicing factor proline/glutamine-rich (SFPQ/PSF), and paraspeckle component 1. Paraspeckles are suggested to regulate gene expression through the nuclear retention of RNAs. In addition to this function, SFPQ/PSF is reportedly involved in DSB repair via canonical non-homologous end joining (NHEJ) and homologous recombination (HR). SFPQ/PSF interacts with DNA and the homologous recombinase Rad51 and TopBP1 proteins. The SFPQ-NONO complex substitutes for XLF, a core c-NHEJ factor, and stabilizes paired DNA DSB ends, resulting in the promotion of DSB repair via NHEJ. The nuclear matrix protein, Martin 3(MATR3), is also associated with the SFPQ-NONO complex. MATR3 was found to be an ATM target, and microbeam-induced DNA damage in MART3-depleted cells led to the abnormal accumulation of S-phase cells; in addition, it was observed that the SFPQ-NONO complex retention at the sites of DNA damage was prolonged in these cells [67,77,78,79,80].

Like SFPQ/MONO proteins, the other paraspeckle proteins FUS, previously mentioned as involved in telomere regulation, and RBM14 are also involved in DDR activation. Both FUS and RBM14 contain an unstructured prion-like domain (PLD). FUS accumulates at laser-induced DSB sites in a PAR-dependent manner. The recruitment of FUS is necessary for γH2AX formation during the DDR and following proper DDR signaling. The interaction between FUS and HDAC1, a chromatin modification enzyme, suggests that FUS is involved in the regulation of histone acetylation following DNA damage and in the modification of chromatin structure during the DNA repair process. Interestingly, FUS proteins that are mutated with familial ALS exhibit diminished FUS-HDAC1 interactions in response to DNA damage [81]. RBM14 is required to recruit XRCC4 and XLF to chromatin and to release KU proteins from chromatin during the DDR. Defects in this process lead to the accumulation of DSBs and prolonged γH2AX foci, suggesting that RBM14 stimulates the DNA repair process by regulating the DNA-PK-dependent NHEJ pathway [82,83].

By genome-wide screening, RBMX (RNA-binding motif protein, X-linked, also known as hnRNP G) was identified as a positive regulator of HR during the DDR. RBMX transiently accumulates at sites of DNA damage and increases the fidelity of DNA end joining, preventing further degradation in a poly(ADP-ribose) polymerase 1 (PARP1)-dependent manner [65,84]. However, PARP-depletion could prevent RBMX accumulation at DNA damage sites without changing the HR efficiency, suggesting that the inhibition of HR after RBMX knockdown might be caused by a reduction in BRCA2 expression. Other hnRNPs, such as hnRNPU-like (hnRNPUL) and hnRNP C, have been identified as components of the MRE11/RAD50/NBS1 (known as the MRN complex) and BRCA1/BRCA2/PALB2 complexes, respectively. These components serve as DNA DSB sensors. hnRNPUL 1 and 2 proteins stimulate DNA-end resection through their recruitment to sites of DNA damage, in an MRN-dependent manner; consequently, DNA repair, initiated by ATR-dependent signaling, proceeds by HR. The authors also showed that hnRNPUL1 and 2 act on downstream of MRN and CtBP-interacting protein, facilitating of the recruitment of the BLM (Bloom syndrome, RecQ helicase-like) helicase to DSBs [72].

PRP19 is known as a core component of the NTC/PRP19 complex, which regulates spliceosome activity through the ubiquitination of PRP3, a component of the U4 snRNP; this leads to stabilization of the U4/U6 and U5 snRNPs [85,86,87,88]. A proteomic screen for proteins that interact with ssDNA-coated with the replication protein A complex identified that PRP19 is also a sensor of DNA damage [89]. PRP19 localizes to sites of DNA damage via RPA, which directly binds PRP19 in vitro. CDC5L, a component of the PRP19 complex, is required for the regulation of signaling downstream of ATR. [90]. Dysfunction of PRP19, specifically loss of binding activity to RPA or ubiquitin E3 ligase activity, results in the inability to support a proper ATR response [89].

## 6. Possible Roles of Non-Coding RNAs in DNA Damage Responses

It has been shown that most of the human genome is transcribed [91]. These numerous transcripts often do not encode proteins, but have certain biologically functional roles. Some of these ncRNAs are localized to the nuclear and might regulate the epigenetic modification of chromatin in a sequence-specific manner [92]. Certain long ncRNAs (>200 nucleotides) have been reported to modulate genotoxic stress responses [93,94,95]. However, most stress-induced ncRNAs have not been shown to directly interact with DNA damage sites during the DDR. Several recent studies demonstrated that RNAs themselves, especially distinct small RNAs, are implicated in the DDR [96,97]. Indeed, RNase treatment attenuates the induction of DNA damage-foci; in addition, small RNA that is locally produced upon DNA damage is required for DNA repair [98]. Although small RNA biogenesis involves various accessory proteins including DGCR8 and other RBPs, in addition to Dicer and Drosha [99], the association between these proteins and DNA damage has not been well studied. Inactivation of Dicer or Drosha leads to the downregulation of DSB-induced small RNA (diRNA). For example, northern blotting, probing for diRNAs and using a probe spanning DSB sites, and RNA deep sequencing have confirmed that these diRNAs are produced from sequences that flank the DSB region in plant and human cells [100]. diRNAs are associated with Argonaute2 (Ago2), and its inactivation decreases the DNA repair efficiency of DSBs. These proteins, which are associated with small RNA biogenesis, are also linked to the DNA repair process through interactions with DDR proteins such as Rad51. In addition to endogenous small RNAs, RNA-elements are used as direct templates for DSB repair. Keskin et al*.* showed that the endogenous transcript-RNA can guide HR at sites of DNA damage in the yeast *Saccharomyces cerevisiae* [101]. Artificial RNA elements can also induce genomic rearrangement. In addition to that in yeast, RNA oligonucleotides can serve as templates for DSB repair of homologous chromosomal DNA in human embryonic kidney 293 cells [102]. Further studies will uncover how RBPs function in the RNA-dependent DNA repair process (Figure 1c).

## 7. RNA Modification and DNA Damage Responses

Xiang et al*.* have provided other evidence suggesting that RNA is directly involved in DNA repair [103]. N6-methyladenosine (m6A) is an RNA modification that modifies the fate and function of the RNA species; the authors identified rapidly-induced m6A RNA modification after the induction of DNA damage by UV laser micro-irradiation in U2OS cells. UV radiation induced the rapid accumulation of m6A on RNA at DNA damage sites marked by γH2A.X histone modification. METTL3, which is m6A methyltransferase, simultaneously localized to sites of DNA damage. The authors also demonstrated that the accumulation and removal of m6A at sites of DNA damage is dependent on the catalytic activity of METLL3 and the m6A demethylase, FTO, respectively. Furthermore, the localization of the DNA repair enzyme, DNA polymerase κ, at DNA damage sites is dependent on the catalytic activity of METTL3, suggesting that m6A modification of RNA after UV irradiation is the initial trigger for the recruitment of DNA repair proteins (Figure 1c).

## 8. Conclusions

Increasing and unexpected roles of RBPs in genome integrity have been recently recognized. Advances in analytical techniques, such as RNA deep sequencing and single cell analysis, have provided far more detailed knowledge of RNA biology. Indeed, many RBPs have the potential to bind DNA, and some are involved in DNA repair or accumulate at sites of DNA damage. In addition to the direct function of RBPs in the DDR, the RNA-mediated DNA repair system is also an interesting mechanism. It is known that lncRNAs are involved in diverse biological processes through their function as scaffolds for molecular interactions. Thus, it will be of interest to further investigate whether lncRNAs are involved in different protein-protein and protein-DNA interactions during DDR activation. Furthermore, recently, RNA modification has been recognized as an important biological process in RNA metabolism and fate. Indeed, the accumulation of m6A modifications at sites of DSBs is proposed to serve as a beacon for the recruitment of DNA polymerase κ for the DNA repair process. Other RNA modifications, such as m1A and m5C, might also contribute to genome integrity through the DNA repair process or R-loop formation. Further investigation of RBPs or RBP-associated RNAs (diRNA, lncRNA, etc.) after DNA damage will provide a deeper understanding of the multilayered maintenance mechanisms governing genome integrity, and help to develop new treatments for the numerous diseases that are linked to DNA damage, such as cancer.
